# Corrigendum to “How to Treat a Signal? Current Basis for RET-Genotype-Oriented Choice of Kinase Inhibitors for the Treatment of Medullary Thyroid Cancer”

**DOI:** 10.1155/2016/2168046

**Published:** 2016-06-28

**Authors:** Hugo Prazeres, Joana Torres, Fernando Rodrigues, Joana P. Couto, João Vinagre, Manuel Sobrinho-Simões, Paula Soares

**Affiliations:** ^1^Cancer Biology Group, Institute of Molecular Pathology and Immunology of the University of Porto (IPATIMUP), Rua Dr. Roberto Frias, s/n, 4200-465 Porto, Portugal; ^2^Molecular Pathology Service, Portuguese Institute of Oncology of Coimbra FG, EPE, Avenida Bissaya Barreto, 98, 3000-075 Coimbra, Portugal; ^3^Department of Pathology, Faculty of Medicine of Porto University, Al. Prof. Hernâni Monteiro, 4200-319 Porto, Portugal; ^4^Endocrinology Service, Portuguese Institute of Oncology of Coimbra FG, EPE, Avenida Bissaya Barreto, 98, 3000-075 Coimbra, Portugal; ^5^Abel Salazar Biomedical Sciences Institute (ICBAS), Lg. Prof. Abel Salazar, 4099-003 Porto, Portugal; ^6^Department of Pathology, Hospital São João, Al. Prof. Hernâni Monteiro, 4200-319 Porto, Portugal

In the article titled “How to Treat a Signal? Current Basis for RET-Genotype-Oriented Choice of Kinase Inhibitors for the Treatment of Medullary Thyroid Cancer” [1], the following sentence should be added to the “Acknowledgments” section: This work was also supported by FEDER funds through Programa Operacional Factores de Competitividade (COMPETE) (FCOMP-01-0124-FEDER 011267).
